# A Computational Model for Investigating Tumor Apoptosis Induced by Mesenchymal Stem Cell-Derived Secretome

**DOI:** 10.1155/2016/4910603

**Published:** 2016-11-09

**Authors:** Melisa Hendrata, Janti Sudiono

**Affiliations:** ^1^Department of Mathematics, California State University, Los Angeles, CA 90032, USA; ^2^Department of Oral Pathology, Faculty of Dentistry, Trisakti University, Jakarta, Indonesia

## Abstract

Apoptosis is a programmed cell death that occurs naturally in physiological and pathological conditions. Defective apoptosis can trigger the development and progression of cancer. Experiments suggest the ability of secretome derived from mesenchymal stem cells (MSC) to induce apoptosis in cancer cells. We develop a hybrid discrete-continuous multiscale model to further investigate the effect of MSC-derived secretome in tumor growth. The model encompasses three biological scales. At the molecular scale, a system of ordinary differential equations regulate the expression of proteins involved in apoptosis signaling pathways. At the cellular scale, discrete equations control cellular migration, phenotypic switching, and proliferation. At the extracellular scale, a system of partial differential equations are employed to describe the dynamics of microenvironmental chemicals concentrations. The simulation is able to produce both avascular tumor growth rate and phenotypic patterns as observed in the experiments. In addition, we obtain good quantitative agreements with the experimental data on the apoptosis of HeLa cancer cells treated with MSC-derived secretome. We use this model to predict the growth of avascular tumor under various secretome concentrations over time.

## 1. Introduction

Apoptosis is a normal, genetically regulated process in which a cell undergoes a sequence of intracellular complex processes that trigger self-destruction. Cancers occur due to mutations of certain fundamental genes that disable the cells to perform apoptosis, giving rise to malignant tumor cells that grow uncontrollably. With its genetic instability, an individual tumor cell becomes a forerunner parent cell that has the potential to develop into a cluster, biologically complex tumor consisting of approximately 10^6^ cells.

Various cancer treatments have been explored with the ultimate goal of suppressing its growth and spreading and perhaps even eradicating cancerous cells. Recently, mesenchymal stem cells (MSCs) have become a topic of great focus in relation to cancer. MSCs are known to secrete a broad panel of proteins including growth factors, chemokines, and cytokines, which are called secretome [1]. Growing evidence suggests that MSCs have an important role in affecting the behavior of tumor cells [2]. While some studies reported that MSCs favor tumor growth, others showed that MSCs can suppress tumorigenesis [3, 4]. In particular, it has been reported that secretome contained in conditioned media (CM) of MSCs promotes apoptosis and autophagy of cancer cells [5]. Experiments done by Sandra et al. show that secretome significantly induces apoptosis in HeLa cancer cells in concentration and time dependent manner [6].

From intracellular perspective, there are two well-known major signaling pathways leading to apoptosis: the intrinsic pathway centered on mitochondria and the extrinsic pathway initiated by death receptors called Tumor Necrosis Factor (TNF). There is now evidence showing that these two pathways are connected and affect one another [7, 8]. Moreover, recent research has also revealed the third pathway, called the perforin pathway, which involves T-cell mediated cytotoxicity and is induced by granzyme B protein. Perforin pathway is also connected to the intrinsic pathway and all three pathways eventually converge into the activation of caspase 3 protein leading to cell death, chromatin condensation, chromosome fragmentation, nuclear degradation, and protein cytoskeleton [8–10].

Understanding the dynamics of secretome-induced apoptosis that can modulate cells' life and death can immensely provide therapeutic potential. Despite numerous experimental studies, the underlying biological mechanism of tumor apoptosis induced by MSC secretome is not yet fully understood. Laboratory experiments may not be cost effective and are often quite challenging to perform as each experiment can only be done for specific cells and cannot be easily modified to investigate others. Computational model that simulates secretome-induced apoptosis provides general virtual solution that could complement experimental methods.

For a long time, various modeling techniques have been used to simulate avascular tumor growth [11–20]. Continuous models consider the interactions between cell density and chemical concentrations that influence cell cycle events of tumor cell population. These models employ a system of partial differential equations to describe reaction-diffusion-convection of cells and their microenvironmental elements. Continuous models are computationally cost effective in general; however, they do not maintain cell-specific properties and individual cell interactions. On the other hand, discrete models such as cellular automata, extended Potts, and agent-based models focus on modeling single-cell phenomena and upscale it to obtain information about macroscopic phenomena of tumor growth. Drawbacks of the discrete approach lie in their parametrization and the computational costs, but it provides greater qualitative insight into the nature of the system. Hybrid discrete-continuous models provide the benefits of both implementations within the same simulation. Some of the models listed above are multiscale models that typically include cellular, subcellular, and extracellular levels. However, all of these models simulated cancer growth in an untreated environment, and hence none of them includes any apoptosis-related signaling network at their subcellular level. Previous modeling work on apoptosis itself, such as [21–23], mostly focused on partial signaling pathways. Hong et al. [24] proposed a continuous ordinary differential equations (ODE) model for the apoptosis signaling network to study the effect of cisplatin. Even though their comprehensive model included three major pathways involved in cisplatin induced apoptosis, namely, the mitochondrial, death receptor-mediated, and endoplasmic reticulum-stress pathways, it is a single scale model at the molecular level and is not integrated to the other levels of the system.

In this study, we develop a multiscale hybrid discrete-continuous model that integrates continuous models of the apoptosis signaling pathways and chemical concentration dynamics at the molecular and extracellular levels into a discrete agent-based model at the cellular level. Our apoptosis signaling pathways model is a system of ordinary differential equations (ODEs) that comprehensively covers all three known pathways that are involved in secretome-induced apoptosis. Our simulation produces phenotypic patterns of avascular tumor growth as observed in the experiments. The model also verifies and obtains a good quantitative agreement with the experimental results by Sandra et al. [6] in studying the role of secretome in inducing apoptosis of HeLa cancer cells. This suggests that the model can potentially be used as a tool in predicting tumor apoptosis induced by various substances. With this model, we further quantify the contribution of each signaling pathway in inducing apoptosis. Lastly, we use the model to predict the effect of secretome of various concentrations on tumor spheroid growth.

## 2. Materials and Methods

Our model spans across three biological time scales: molecular scale, cellular scale, and extracellular scale, which are closely integrated. At the molecular scale, the apoptosis signaling network regulates cellular apoptosis induced by secretome. At the cellular level, a discrete agent-based model controls cell migration, proliferation, and death. At the extracellular level, a system of partial differential equations describes diffusion, consumption or production, and decay of extracellular substances, such as nutrient (oxygen), extracellular matrix, matrix-degradative enzyme, and growth inhibitors.

### 2.1. Molecular Scale: Apoptotic Signaling Pathways

Literature study has shown that the three major signaling pathways that are known to be involved in apoptosis are the extrinsic (death receptor) pathway, intrinsic (mitochondrial) pathway, and the perforin pathway [8–10]. The extrinsic and intrinsic pathways we use here are adopted from various sources [8, 10, 24–26] with minor modifications. We integrate the perforin pathway in order to build a comprehensive model that covers all signaling pathways known to be involved in apoptosis induced by MSC secretome. When a cell detects nonzero concentration of the secretome in the medium, a cascade of molecular events occur along these pathways.

**Figure 1:**
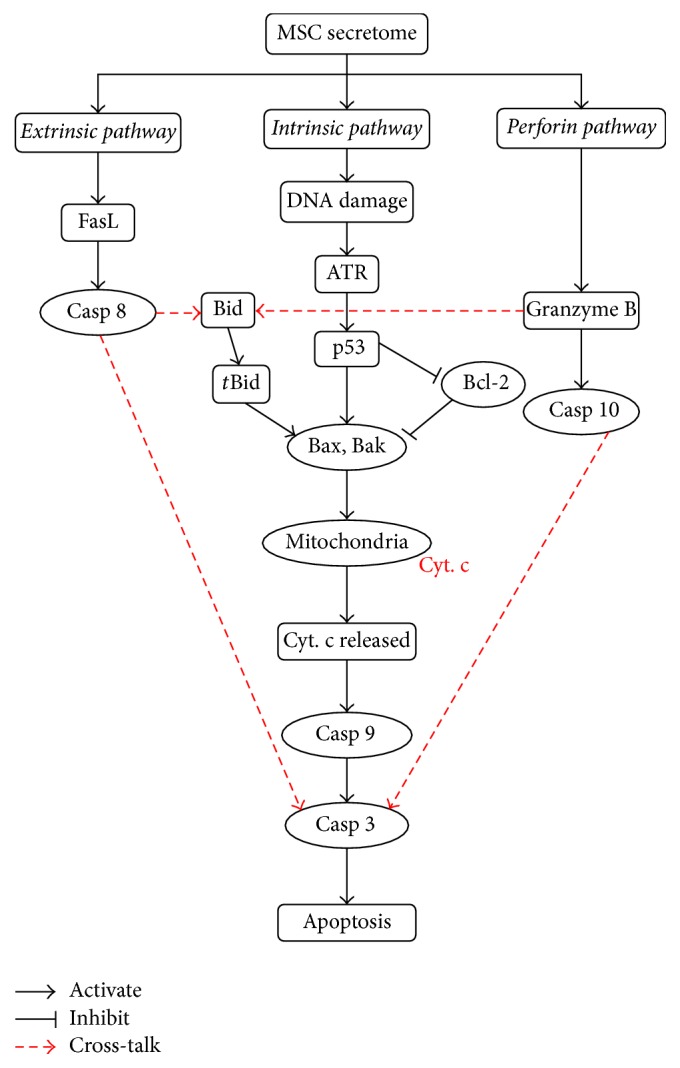
A schematic model of the three apoptosis signaling pathways. (1) The extrinsic pathway mediated by death receptors, (2) the intrinsic pathway centered on mitochondria, and (3) the perforin pathway induced by granzyme B. Each pathway activates its own initiator caspase (casp 8, 9, and 10) which in turn will activate the executioner caspase 3. A solid arrow indicates activation or upregulation, while a line terminated by a bar indicates inhibition or downregulation. The arrows with broken red lines indicate the cross-talk between these pathways.

A schematic model of the pathways used in this paper is shown in Figure 1. The intrinsic pathway begins as secretome induces DNA damage, which further results in the activation of ATR and p53 proteins. As a response to the DNA damage, the proapoptosis proteins, such as Bax and Bak, will be activated, leading to the opening of mitochondrial permeability transition pore. This triggers the release of cytochrome c from mitochondria into the cytosol [27–30]. On the other hand, the antiapoptosis protein, such as Bcl-2, will inhibit the release of cytochrome c. Cytochrome c will bind with Apaf-1 and activate caspase 9. Activated caspase 9 will then cleave and activate downstream caspases, such as caspase 3, which is also known as the apoptosis executor protein.

The extrinsic pathway is initiated by death receptors, called Tumor Necrosis Factor (TNF). The binding of TNF to its receptor causes the level of FasL to increase, which leads to the downstream activation of caspase 8. Activated caspase 8 can trigger the intrinsic pathway through the cleavage of Bid. The truncated Bid further stimulates Bax and Bak. Alternatively, the activated caspase 8 can bypass the intrinsic pathway by directly initiating the activation of caspase 3 [8, 31].

The perforin pathway involves T-cell mediated cytotoxicity and is perforin-granzyme dependent in activating caspase 10, which subsequently triggers the activation of caspase 3. The interconnection (cross-talk) between the perforin and intrinsic pathways occurs through the truncation of Bid by the activated granzyme B [8]. All three pathways eventually merge on the activation of caspase 3 that induces cellular apoptosis.

The change of concentration of each protein involved in the signaling pathways over time is given by an ordinary differential equation (ODE) of the form

(1) $$\begin{array}{c} \frac{d\left[C\right]}{dt}=\sum k_{1}\left[\;S_{1}\;\right]-\sum k_{2}\left[\;S_{2}\;\right], \end{array}$$

where [*C*] is the concentration of the chemical, *k*_1_[*S*_1_] is the production rate, and *k*_2_[*S*_2_] is the consumption rate of [*C*]. The biochemical kinetics involved in the model in Figure 1 are given in Table 1 and their corresponding system of ODEs are listed in Table 2. Blocks A, B, and C in Table 2 list the equations involved in extrinsic, intrinsic, and perforin pathways, respectively. Block D in this table lists the equations that are needed by all three pathways.

Since it is assumed that secretome is distributed uniformly across the simulation domain, each cell initially detects the same level of secretome and solves the system of ODEs to determine its apoptosis level at each time step. In our simulation, we employ the classical fourth-order Runge-Kutta method to solve the system numerically [37]. Reaction rate constants and the apoptosis proteins' initial values used in our simulation are listed in Tables 3 and 4.

### 2.2. Extracellular Scale: Reaction-Diffusion for Biochemical Concentration

Cells interact and respond to their microenvironment, which is characterized by local extracellular biochemical concentration. Combining the models proposed by [16, 17, 19], we employ reaction-diffusion equations to model the dynamics of these chemicals, which include nutrient concentration *u*, waste (growth inhibitor) concentration *w*, extracellular matrix (ECM) density *f*, and matrix-degradative enzyme (MDE) concentration *m*. Each of these quantities is a function of spatial variable **x** and temporal variable *t*.

#### 2.2.1. Nutrient (Oxygen) Concentration

In our model, local nutrient concentration is one of the key factors that determines cell's viability, aside from cellular apoptosis. At the macroscopic scale, the evolution of nutrient concentration *u*(**x**, *t*) is given by the following reaction-diffusion equation:

(2) $$\begin{array}{c} \frac{\partial u}{\partial t}=D_{u}\nabla^{2}u-\overset{N\left(t\right)}{\underset{k=1}{\sum }}\gamma_{u}\left(\;x_{k}\;\right)e^{-{\left|x-x_{k}\right|}^{2}/\epsilon^{2}}-\delta_{u}u, \end{array}$$

where *D*_*u*_ is the nutrient diffusion constant, *N*(*t*) is the number of cells at time *t*, *γ*_*u*_(**x**_*k*_) is the nutrient consumption rate of cell *k* which depends on the cell's viability status and its position **x**_*k*_, *ϵ* is the degree of localized nutrient consumption, and *δ*_*u*_ is nutrient decay rate. The first term of the equation represents the diffusion of the nutrient in the medium, while the summation in the second term defines the total nutrient uptake by tumor cells that are still viable or quiescent. Necrotic and apoptotic cells do not consume any nutrients. At any given point** x** in the medium, individual cell's nutrient uptake rate is a function of cell's position and it declines exponentially as distance increases. Data from [38, 39] show that the nutrient uptake rate of quiescent cells is approximately half that of proliferating cells. For necrotic and apoptotic cells, the uptake rate *γ*_*u*_ is set to 0. Cell *k*'s local nutrient concentration is the value *u*(**x**, *t*), where **x** is the nearest grid point to the cell's position **x**_*k*_. We define two threshold values *T*_1_ and *T*_2_ for the cell's local nutrient concentration to determine its viability status. Cell *k* is said to be viable whenever *u*(**x**, *t*) > *T*_2_, quiescent if *T*_1_ ≤ *u*(**x**, *t*) ≤ *T*_2_, and necrotic otherwise. As oxygen is one of the most important cellular nutrients that is crucial for cell metabolism, we chose the parameter values of diffusion constant *D*_*u*_, consumption rate *γ*_*u*_, and decay rate *δ*_*u*_ to be those of oxygen.

#### 2.2.2. Matrix-Degradative Enzyme (MDE) Concentration

The equation for MDE concentration *m*(**x**, *t*) is very much similar to the reaction-diffusion equation for nutrient concentration discussed above with the exception that active MDE is produced by cells and decays at a constant rate.

(3) $$\begin{array}{c} \frac{\partial m}{\partial t}=D_{m}\nabla^{2}m+\overset{N\left(t\right)}{\underset{k=1}{\sum }}\mu e^{-{\left|x-x_{k}\right|}^{2}/\epsilon^{2}}-\delta_{m}m. \end{array}$$

Here the parameters *D*_*m*_, *δ*_*m*_, and *μ* are the MDE diffusion coefficient, decay rate, and single cell MDE production rate, respectively.

#### 2.2.3. Extracellular Matrix (ECM) Density

Cells adhere to the extracellular matrix (ECM) and require the ECM for certain types of cell movement. In this model, we assume that ECM consists of only macromolecules, such as laminin, fibronectin, and collagen, and does not contain any other cells. These macromolecules are known to be important for cell adhesion, spreading, and motility and they are bound to the surrounding tissue. Moreover, tumor invasion and metastatic process depend on the cell's ability to degrade the ECM [40–42]. The tumor cells produce MDE which degrade the ECM locally upon contact and the degradation process is modeled by the following equation:

(4) $$\begin{array}{c} \frac{\partial f}{\partial t}=-\delta_{f}mf, \end{array}$$

where *δ*_*f*_ is the degradation rate, *m* is the MDE concentration, and *f* is the ECM density.

#### 2.2.4. Growth Inhibitor Concentration

Growth inhibitory factors, such as waste products and lactate, are released by necrotic cells into the medium and diffuse outward from the center of the tumor mass. The production and diffusion of inhibitory factors are computed similarly as in MDE concentration (3), except that the production is computed over necrotic cells only.

(5) $$\begin{array}{c} \frac{\partial w}{\partial t}=D_{w}\nabla^{2}w+\overset{\hat{N}\left(t\right)}{\underset{k=1}{\sum }}\alpha e^{-{\left|x-x_{k}\right|}^{2}/\epsilon^{2}}, \end{array}$$

where $\hat{N}(t)$ is the total number of necrotic cells at time *t* and *α* is the production rate of growth inhibitor by a single necrotic cell. As growth inhibitor diffuses outward from the tumor, it will eventually reach viable cells in the outer rim. When the accumulated growth inhibitor reaches a certain threshold value *T*_3_, viable cells undergo proliferation arrest and become quiescent even though the cells' local nutrient concentration is still sufficiently high.

Our simulation uses parameter values that are derived from experiments as much as possible. We estimate parameter values whose data are not available to achieve best possible agreement with experimental results. The list of parameters used in (2)–(5) and their references are listed in Table 5.

### 2.3. Cellular Scale: Motility and Phenotypic Switching

In our two-dimensional agent-based model, each tumor cell has a fixed radius *r* with individual cell data consisting of cell position, viability status, nutrient consumption rate, and cell proliferation clock. These individual data are stored and updated at each time step. The discrete component of the model regulates individual cell processes such as cell growth and proliferation, as well as cellular adhesion and interactions that play important roles in cell migration.

#### 2.3.1. Cell Migration

Every tumor cell is treated as an autonomous agent that updates its position according to the discrete equation:

(6) $$\begin{array}{c} x_{k}\left(\;t+\Delta t\;\right)=x_{k}\left(\;t\;\right)+vV_{k}\left(\;t\;\right)\Delta t, \end{array}$$

where Δ*t* is the time step, **x**_*k*_(*t*) is the position of cell *k* at time *t*, *v* is the step length, and **V**_*k*_(*t*) is the motility direction of cell *k* at time *t*. Each cell is subject to competing forces that determine its direction of motion within the microenvironment. In this model we consider two of such forces: intercellular adhesion and cell-ECM adhesion, which is sometimes referred to as haptotaxis. The direction of movement **V**(*t*) is their weighted average:

(7) $$\begin{array}{c} V\left(\;t\;\right)=d_{I}V_{I}\left(\;t\;\right)+d_{H}V_{H}\left(\;t\;\right). \end{array}$$

Here the subscripts *I* and *H* denote the intercellular adhesion and haptotaxis, respectively, and *d* is the weight of the velocity due to each biasing agent. Since there are no exact known macroscopic forces that govern cellular adhesion and haptotaxis, we implement some force formulas that have been conventionally used in biophysical models as described below.

The intercellular forces take into account both cell-cell adhesion and repulsion. This pairwise interaction between two interacting bodies is modeled via the potential function *U* as defined in [17, 43]

(8) $$\begin{array}{c} U_{k,j}=C_{A}e^{-\left|x_{k}-x_{j}\right|/l_{A}}-C_{R}e^{-\left|x_{k}-x_{j}\right|/l_{R}}. \end{array}$$

The first term of the above equation gives the adhesion term and the latter specifies the repulsion term between two distinct cells *k* and *j*. It also assumes only pairwise interactions and ignores *N*-body interactions for *N* > 2. The parameters *C*_*A*_, *C*_*R*_ define the adhesion and repulsion strengths, respectively, and *l*_*A*_, *l*_*R*_ their effective length scales [44]. The velocity direction due to cell-cell adhesion forces for cell *k* is determined by the sum of the interaction potential gradients from all other cells as follows:

(9) $$\begin{array}{c} V_{I}^{k}=\overset{N\left(t\right)}{\underset{j=1,j\neq k}{\sum }}\nabla U_{k,j}, \end{array}$$

where *U*_*k*,*j*_ is the potential force between cells *k* and *j*.

Haptotaxis is defined as a directed migratory response of cells to gradient of fixed nondiffusible chemicals. Studies have been done to characterize such directed movement in tumor cells [42, 45, 46] and it was found that migrating cells choose pathways with the highest availability of ECM proteins, such as fibronectin. In our model, haptotaxis movement is specifically defined to be the upward movement of cells along the gradients of bound extracellular matrix:

(10) $$\begin{array}{c} V_{H}^{k}=\xi \nabla f\left(\;x_{k}\;\right) \end{array}$$

with *ξ* denoting the haptotaxis coefficient.

#### 2.3.2. Cell Proliferation

Cell proliferation cycle consists of the growth phase and division phase. DNA synthesis occurs during the growth phase, while cytokinesis takes place during division phase. A cell divides after both have been completed. Five checks are performed on cells prior to performing mitosis:

(1) *Viability Status*. Only viable cells have a chance to perform mitosis. This is based on the reasoning that cellular energy is prioritized for basal metabolism needed for cell survival, and hence cell growth slows down or even stops when it senses nutrient shortage.
(2) *Proliferation Age*. A cell must reach a certain age to ensure it has enough time to complete all stages of the cell cycle. In our model, each cell is assigned a cell clock with a random phase, which ticks to a maximum time *T* that corresponds to the duration of a cell cycle. When the cell clock reaches *T*, a cell matures and the next check (space availability check) is performed.
(3) *Space Availability*. Mitosis is allowed if there is sufficient space around the parent cell for the two new daughter cells to occupy. To check this condition, we adopt a method used in [17] by examining the cell's repulsion term from the interaction potential equation (8). Cell division is allowed only if the total repulsion force of the cell falls below a predefined constant. Otherwise, the cell enters quiescent state.
(4) *Growth Inhibitor Level*. Viable mature cells whose local concentration of growth inhibitor is above a threshold value cannot proliferate and they become quiescent.
(5) *Cell Shedding*. The last check on cell shedding is based on experimental observation that mitotic cells are lost from tumor spheroid surface at a constant rate per spheroid surface, that is, 20.9 ± 1.0 cells per sq mm of spheroid surface per hour [36]. For simulation purpose, we let a mitotic cell on the outermost part shed away from the tumor with 20% probability.

When all conditions are met and a cell does not shed, mitosis is performed. Cell division is modeled by having one daughter cell replace the parent cell, and the second daughter cell takes a small random offset from the first cell's position.

The list of parameters used in the discrete part (cellular level) of the model is listed in Table 6.

## 3. Results

We implement the model proposed above in an off-lattice two-dimensional setting. The off-lattice agent-based model is chosen to reduce geometric constraint and artificiality, while two-dimensional algorithm implementation reduces its computational costs. Each cell is equipped with its individual cellular properties, for example, cellular phenotype (viability status), age, and its apoptosis signaling pathways. The cell moves according to its local interaction with other cells (adhesion and repulsion) and its surrounding extracellular matrix (haptotaxis).

**Figure 2:**
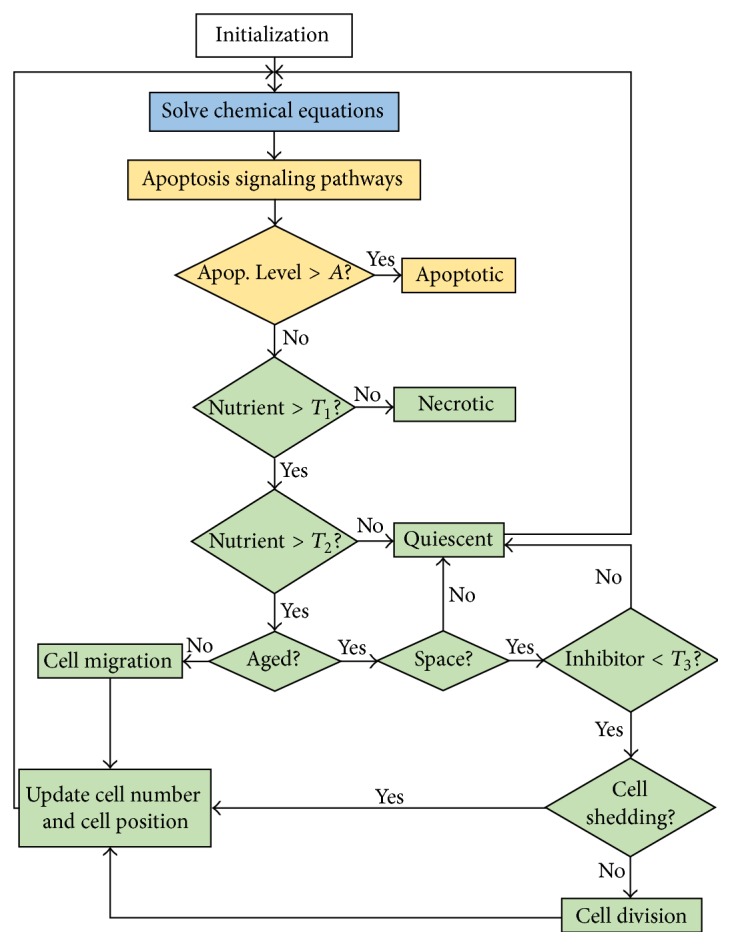
Flowchart showing the integration of molecular, cellular, and extracellular scales into a sequence of events executed at each iteration. The molecular level processes are shown in yellow, extracellular level process is shown in blue, and cellular level processes are shown in green.

The integration of the molecular, cellular, and extracellular time scales and the sequence of steps computed by a cell at each iteration are illustrated in the flowchart in Figure 2. In the flowchart, the molecular level processes are colored in yellow, the extracellular process in blue, and the cellular level processes in green.

We run several sets of simulations for various purposes. The first set of simulations is done to test the accuracy of our proposed apoptotic signaling model at the molecular level (Tables 1 and 2). The second set of simulations tests the model at cellular and extracellular levels. Here we implement the algorithm defined by (2)–(10) without the apoptosis signaling network and compare the simulation result with experimental result on tumor spheroid growth. In the last set of simulations, we integrate the apoptotic signaling network into tumor growth simulation to predict the effect of secretome in tumor spheroid growth.

### 3.1. Apoptosis Signaling Network Simulation

#### 3.1.1. Baseline Result

Following the laboratory experiment performed by Sandra et al. [6], we place a monolayer consisting of 2 · 10^5^ cells in a nutrient-free secretome-conditioned medium. These cells are spread uniformly across the simulation domain, initially viable with their initial apoptosis level set to 0. The secretome concentration is homogeneous across the domain; hence all cells in one particular simulation detect the same level of secretome concentration. Each individual cell computes its biochemical kinetics equations of apoptotic signaling pathways in Table 2 to determine its apoptosis level over time.

In experiments, a feasible way to measure the effect of secretome in inducing apoptosis is by counting the number of apoptotic cells under different secretome concentration over time. To compare the simulation result with these experimental data, we need to first determine a threshold value *A* that sets the cell to become apoptotic once its apoptosis level passes this threshold value. Since this value is not available in literature, we estimate it through repeated simulations. We test a sequence of values for *A* in the increment of 0.05 starting from 0.05 up to 1. That is, let *A*_*k*_ = 0.05*k*,  *k* = 1,2,…, 20. For each *A*_*k*_, we run the simulation and compute the sum of the square errors *E*_*k*_. The error is defined to be the difference between experimental data and simulation result for each secretome concentration (0.2%, 2%, and 20%) under 24- and 48-hour treatments:

(11) $$\begin{array}{c} E_{k}=\overset{2}{\underset{i=1}{\sum }}\overset{3}{\underset{\;j=1}{\sum }}{\left(\;S_{ij}-D_{ij}\;\right)}^{2}, \end{array}$$

where *j* = 1,2, 3 indicates secretome concentration of 0.2,2 and 20%, respectively, *i* = 1,2 represents the treatment time of 24 and 48 hours, *S*_*ij*_ is the percentage of the apoptotic cells under *j* secretome concentration during *i* treatment time obtained from simulation, and *D*_*ij*_ is the corresponding experimental data. The value of *A*_*k*_ that gives the least square error *E*_*k*_ is then taken to be the apoptosis threshold *A*. The apoptosis threshold we found is *A* = 0.7. This value can be refined further by taking increment that is smaller than 0.05 for *A*_*k*_.

**Figure 3:**
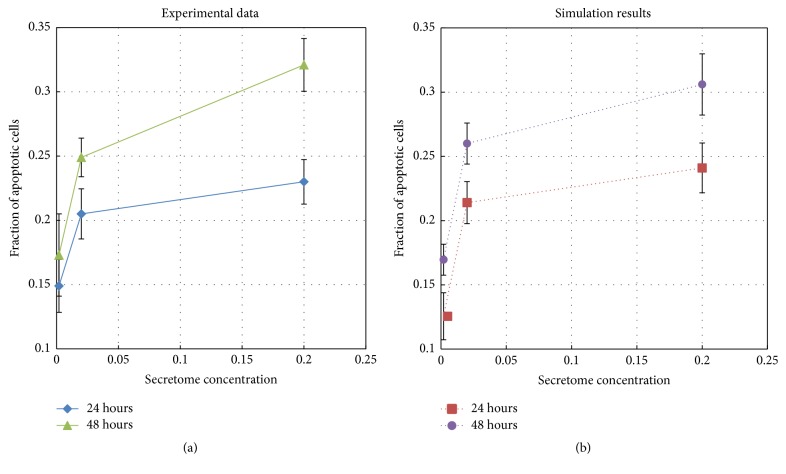
Fraction of apoptotic cells obtained from simulations (dotted lines) and from experimental data (solid lines) for 24 and 48 hours.

We run the simulations with secretome concentration varied from 0.2%, 2%, and 20% under 24- and 48-hour treatments, giving a total of six simulation scenarios. We run each simulation scenario 100 times using the apoptosis threshold value *A* = 0.7. The mean and standard deviation are computed and they are plotted as simulation data point and error bar in Figure 3(b).

This figure shows a fairly good qualitative and quantitative agreement. The apoptosis level monotonically increases as secretome concentration increases. It also increases as period of treatment increases. By comparing the two charts on Figure 3 we can see that the fraction of apoptotic cells produced by the simulation is accurate to within 2.35% for 24 hours and 1.5% for 48 hours treatment for all three secretome concentration levels. This agreement indicates the accuracy and predictive potential of our proposed apoptosis signaling model.

#### 3.1.2. Analysis of Individual Pathway and Cross-Talk Effect

**Figure 4:**
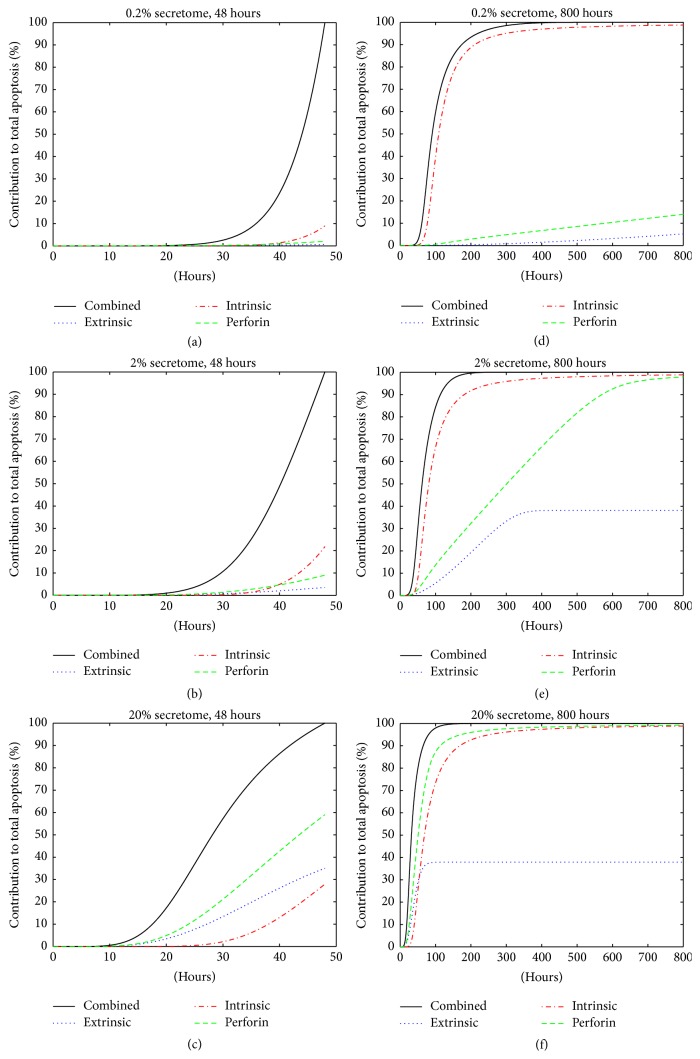
Contribution of individual pathways to the total apoptosis level (in percent). “Combined”: all three pathways are activated; “Extrinsic”: only extrinsic pathway is active; “Intrinsic”: only intrinsic pathway is active; “Perforin”: only perforin pathway is active.

One advantage of having a computer simulated model is that we could measure biological system properties that are hard to quantify in laboratory experiments. One example is quantifying the contribution of each pathway in inducing apoptosis. For this purpose only, on those proteins described in Table 4 as random between [0,1], we intentionally set them equal to 1, while the others stay at 0. This removes the random effect from the initial conditions. The apoptosis (Apop) value obtained from computing all biochemical kinetics equations in Table 2 gives the total apoptosis level from these three pathways combined. To measure the contribution of an individual pathway, we set the other two pathways inactive by assigning their proteins' initial values to 0. For instance, by setting the initial values of FasL, Casp8, granB, and Casp10 to 0 and computing only those equations in blocks B and D of Table 2, we turn off the extrinsic and perforin pathways and hence obtain the apoptosis level contributed by the intrinsic pathway only. In a similar manner, one can measure the apoptosis level produced by extrinsic and perforin pathways separately.  Figure 4 shows the percent contribution of each signaling pathway under different secretome concentration for short term (48 hours) and long term (800 hours) treatment.

We observe the following: First, the apoptosis level contributed by each individual pathway is strictly positive (between 0 and 100%), indicating that all three pathways play roles in cellular apoptosis. Second, in low secretome concentration (0.2% and 2%) the intrinsic pathway gives the highest contribution to the total apoptosis level; see Figures 4(a), 4(b), 4(d), and 4(e). At high secretome concentration (20%), the perforin pathway contributes the highest; see Figures 4(c) and 4(f). This is the case for both 48- and 800-hour treatment period. Third, the contribution from the intrinsic pathway eventually saturates at the level that is very close to the total apoptosis level from all three combined pathways regardless of the secretome concentration (Figures 4(d), 4(e), and 4(f)). This suggests that intrinsic pathway alone can eventually produce the same level of apoptosis, indicating its effectiveness. Lastly, for low secretome concentration (0.2% and 2%) during 48-hour treatment, we notice that the sum of the apoptosis levels from three individual pathways is much less than 100% (Figures 4(a) and 4(b)). This indicates that there is a cross-talk effect between these pathways that gives higher apoptosis level when all three pathways are active. This cross-talk effect seems to decrease as secretome concentration increases, as shown in Table 7, and also as treatment period increases (Figures 4(d), 4(e), and 4(f)). In all cases, the apoptosis level from a single pathway is always less than the apoptosis level generated when several pathways are activated simultaneously.

#### 3.1.3. Sensitivity Analysis

**Figure 5:**
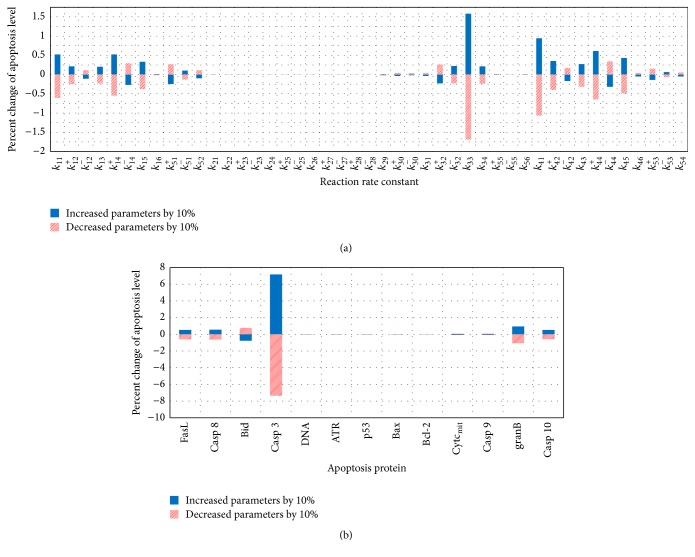
Sensitivity analyses of reaction rate constants (a) and initial conditions of apoptosis proteins (b).

Sensitivity analysis is performed to determine which parameters are most sensitive and whether the system is stable under small perturbations to these sensitive parameter values. We measure the percentage change in the apoptosis level when reaction rate constants and initial value of apoptosis proteins are increased or decreased by 10% from their original values.  Figure 5 shows that the reaction rate constant *k*_33_ and the initial value of caspase 3 protein are the most sensitive parameters in the model. The change in the initial value of caspase 3 by 10% affects the apoptosis level by 7.4%, while the change in reaction rate constant *k*_33_ by 10% causes less than 1.75% change in apoptosis level. This analysis demonstrates the overall robustness of the signaling pathway model.

### 3.2. Avascular Tumor Growth Patterns without Secretome Treatment

In the second set of simulations, we employ (2)–(10) and their corresponding parameter values at the cellular and extracellular levels to model tumor growth without the presence of secretome. In comparison to tumor spheroid that is commonly used as a model system, the two-dimensional simulation results presented here could be interpreted as the cross section through the center of a three-dimensional tumor spheroid.

Our simulation domain is a square with length 1 mm. To implement the extracellular scale, we divide the domain into grids with uniform size *dx* = *dy* = 0.005. Equations (2)–(5) are solved numerically by using the finite difference method [47]. Homogeneous Neumann boundary conditions for the PDEs are applied by assuming zero flux along the domain boundary. The choice for this type of boundary condition is based on the assumption that the nutrient (oxygen), MDE, extracellular matrix, and growth inhibitor remain within this domain. At the cellular scale, each cell is equipped with a proliferation clock that functions as a periodic timer to keep track of the cell's proliferation. In this set of simulation, the algorithm executes all steps shown in the flowchart in Figure 2, except those processes at the molecular level (colored in yellow).

The simulation captures tumor development from a single cell at *t* = 0 up to more than 10,000 cells at *t* = 20 days. It initially starts with a single viable tumor cell with its cellular parameters set according to values in Table 6. The cell is placed in the center of simulation domain. In our simulation, cell typically divides every 10 iterations, which is equivalent to 0.8 days. This is the average doubling time for HeLa cells in suspension [36]. Hence, one time step in our simulation corresponds to 2 hours. At each iteration, we first solve the reaction-diffusion equations (2)–(5) for the microenvironmental chemicals for 2 hours to obtain their current concentrations. From its local nutrient concentration, cell determines whether it stays viable, or becomes quiescent or necrotic. Next, the viable cell checks its proliferation clock to determine if it has acquired certain growth and has aged enough to proliferate. If it has not, the cell computes its intercellular forces to determine its direction of movement for the next iteration. On the other hand, if a cell has matured, it performs a sequence of checks (age, space, inhibitory factor concentration, and cell shedding) as described in Section 2.3. At this point, a viable cell can either divide into two cells, gets shed away from the surface of the spheroid, or becomes quiescent. From the flowchart, we can see that quiescent state is reversible, meaning that a quiescent cell can still become viable, while necrotic state is irreversible.

**Figure 6:**
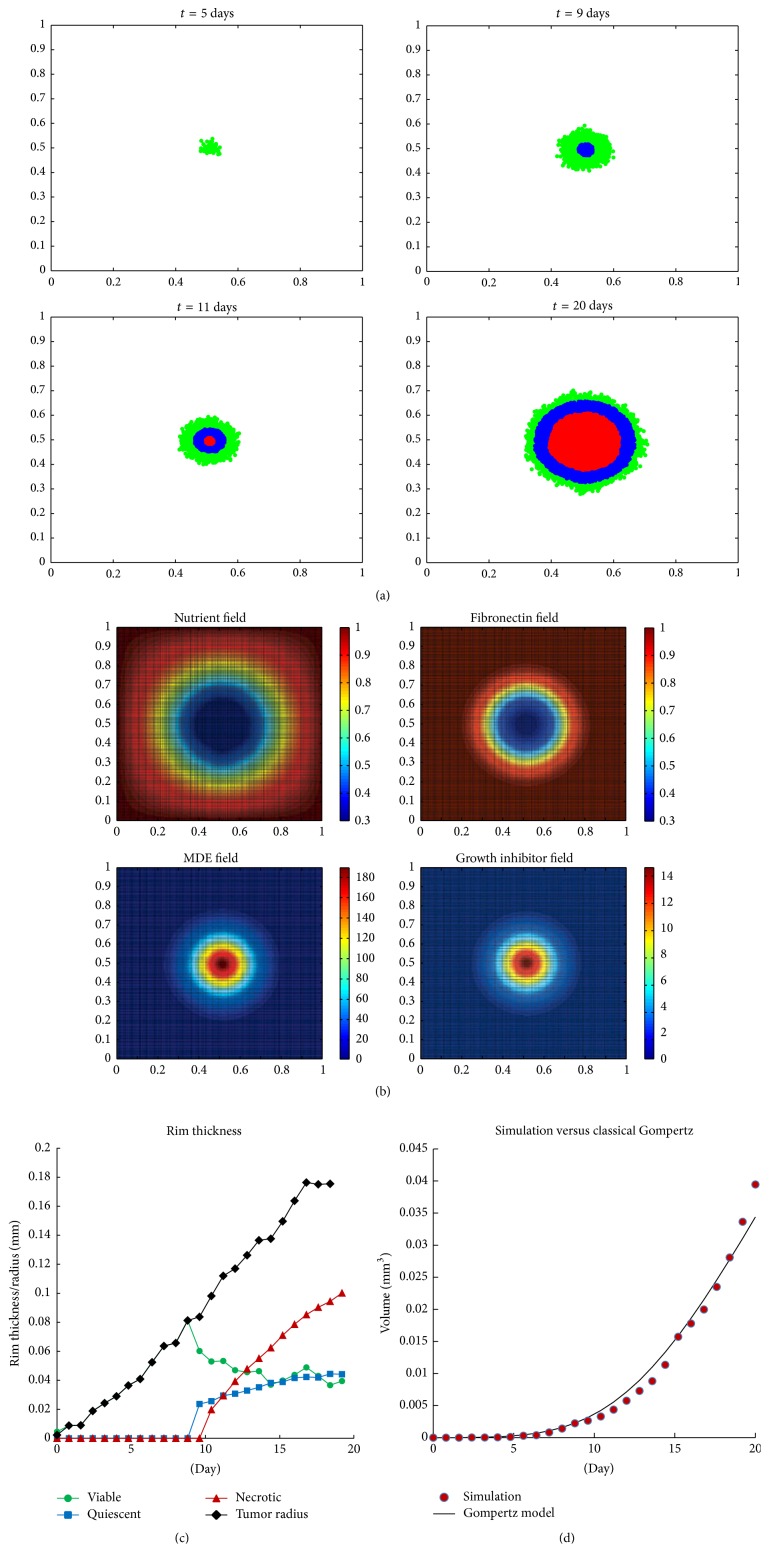
Simulation result of avascular tumor development without secretome. (a) The evolution of tumor growth patterns at *t* = 5,9, 11,20 days. Red: necrotic cells; blue: quiescent cells; green: viable cells. (b) The distribution of microenvironmental factors (nutrient, fibronectin, MDE, and growth inhibitory factors) at *t* = 20 days. (c) The thickness of viable and quiescent rims in comparison with the necrotic core and tumor radii during the first 20 days. ⧫: tumor radius; •: thickness of viable rim; ■: thickness of quiescent rim; ▴: necrotic core radius. (d) The volume as function of time fitted with Gompertz model. The circles are the simulation result and the solid curve is Gompertz curve with parameters *A* = 1.13 and *B* = 0.11. These parameter values are found using the least square technique.

Our simulations are able to produce avascular tumor growth pattern as observed in the spheroid experiment, as well as a quantitative agreement in the growth rate with the growth rate given by classical Gompertz model.  Figure 6(a) shows avascular tumor evolution patterns. All cells are viable (colored in green) during the first 8 days as the nutrient can still diffuse through the entire tumor enabling the cells to maintain their viability. However, as the number of cells is growing, the total nutrient uptake becomes higher than its diffusion rate. The center core becomes nutrient deprived and after day 8, quiescent cells (colored in blue) start to appear. The appearance of necrotic core (colored in red) follows shortly after day 10. The tumor morphology consisting of three layers of viable, quiescent, and necrotic regions is maintained as the tumor diameter increases over time.  Figure 6(b) shows the distributions of nutrient, fibronectin, MDE, and growth inhibitory factors at *t* = 20 days.  Figure 6(c) shows the size of viable and quiescent rim thickness as well as the diameter of the necrotic core during tumor evolution. Starting on day 9, the size of viable rim (shown in green) drops and the thickness of quiescent rim (shown in blue) and necrotic core radius (shown in red) start to increase. From day 10 onward, the necrotic core radius grows as the tumor continues to grow. On the other hand, the thickness of viable and quiescent rims seem to stabilize at approximately 0.04 mm as the proliferation rate of the viable cells on the outer layer is balanced out with the rate of nutrient depletion on the inner part of spheroid that causes viable cells to become quiescent.

We further test the model quantitatively by comparing the growth kinetics of tumor in our simulation with the classical Gompertz model:

(12) $$\begin{array}{c} V\left(\;t\;\right)=V_{0}\exp\left[\;\frac{A}{B}\left(\;1-\exp\left(\;-Bt\;\right)\;\right)\;\right], \end{array}$$

where *V*_0_ is the initial volume in mm^3^ and *V*(*t*) is the volume at time *t* (in days). The parameters *A* and *B* denote the growth and retardation parameters. Since our simulation result only shows two-dimensional cross section through the center of the tumor, we compute an estimate of tumor spheroid volume by the relation *V* = (4/3)*π*(*d*/2)^3^, where diameter *d* is measured by taking the longest distance between any two cells in the tumor. Using the least square method, we found the parameters *A* ≈ 1.13 and *B* ≈ 0.11. See Figure 6(d). These estimates are comparable with the numbers obtained by Sasaki et al. in their spheroid experiments consisting of HeLa cells alone [36], where they found that *A* ≈ 1.23 and 0.9 at lower and higher cell density, respectively, and *B* ≈ 0.1 in both densities.

### 3.3. Tumor Growth under Secretome Treatment

In the next simulation, we integrate the molecular level (apoptosis signaling model) into the tumor growth model. Each cell is now additionally equipped with its apoptosis signaling pathway and their proteins are set according to values in Table 4. Given the preset amount of secretome concentration, each viable or quiescent cell undergoes a cascade of molecular events described by the system of ODEs in the apoptosis signaling pathways (Table 2). The cell's current apoptosis level is determined by solving the system. We set the apoptosis threshold value to be equal to *A* = 0.7 as done previously.

**Figure 7:**
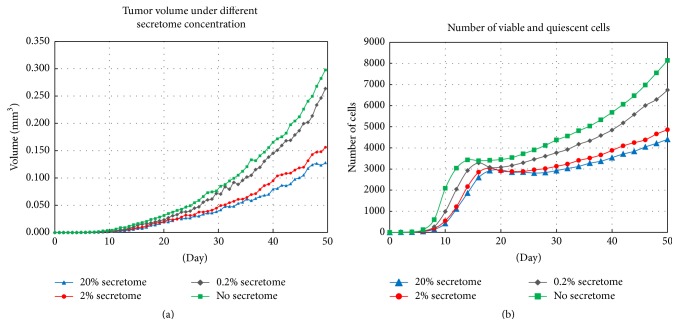
Simulation result of avascular tumor development with various concentrations of secretome up to *t* = 50 days. (a) Comparison of tumor volume treated without secretome (green) and with 0.2% secretome (black), 2% secretome (red), and 20% secretome (blue). (b) Comparison of the number of live cells in untreated tumors and those treated with 0.2%, 2%, and 20% secretome.

We run the simulations with secretome concentrations of 0% (no secretome), 0.2%, 2%, and 20%. Tumor diameter during the first 50 days of the development is then measured and the volume is estimated by applying the formula *V* = (4/3)*π*(*d*/2)^3^, where *d* is the diameter of the tumor.  Figure 7 shows that secretome affects tumor growth in concentration dependent manner. During the first 10 days, there is no significant difference in volume between the untreated tumors and those treated with secretome. Starting day 11, the difference becomes more prominent with tumor treated with 20% secretome only grows up to 0.128 mm^3^, while the untreated tumor grows up to 0.30 mm^3^ at the end of 50-day period (see Figure 7(a)). This shows that 20% secretome concentration can effectively suppress tumor growth by approximately 57%.

We also calculate the number of live cells during the first 50 days. Both viable and quiescent cells are considered as live cells since quiescent cells can still return to viable state.  Figure 7(b) shows that tumor treated with 20% secretome concentration has the lowest number of live cells (4404 cells), followed by tumor with 2% secretome concentration (4860 cells), 0.2% (6741 cells), and the one without secretome treatment has 8143 live cells.

## 4. Discussion

Understanding the mechanism of apoptosis signaling pathways is important in predicting tumor growth under apoptosis-inducing substances, such as MSC-derived secretome. To achieve this goal, we develop a multiscale model that integrates apoptosis signaling pathways with cellular interaction and extracellular microenvironmental dynamics. With this model, we run three sets of simulations to test each level of the model against known experimental and literature data and gain further insight into the underlying processes of the system.

The first set of simulations is run to test the apoptosis signaling pathway model at the molecular level. The simulation shows that higher level of secretome concentration or longer period of treatment causes higher number of cells to undergo apoptosis. This result is supported by the observations of Sandra et al. [6] in their experiments with HeLa cancer cells and shows a good quantitative agreement with their data. We compare the apoptosis level obtained by a single pathway and the one obtained when all three pathways are running concurrently. Our simulation shows that, among three signaling pathways, the intrinsic pathway gives the greatest contribution to the apoptosis level in low secretome concentrations (e.g., 0.2% and 2%), while perforin pathway contributes the highest when the secretome concentration is fairly high (e.g., 20%). Moreover, the intrinsic pathway alone can produce apoptosis level that approaches the apoptosis level produced when all three pathways are active simultaneously. Even though this result suggests the effectiveness of the intrinsic pathway in inducing apoptosis, further experimental studies and model analysis are needed to confirm this. The sensitivity analysis reveals sensitive parameters in the signaling pathway model and confirms that the model is relatively robust and stable under fluctuations of these parameters.

In the second set of simulations, we test our algorithm for the cellular interaction and the PDE model for the microenvironmental dynamics. The apoptosis signaling pathways are omitted in these simulations so that we can analyze the avascular growth without secretome treatment. The simulation is able to reproduce the concentric pattern of necrotic, quiescent, and viable regions of tumor cells during avascular growth as observed in tumor spheroid experiments. Using parameter values of HeLa cancer cells, our simulation result of tumor volume very much agrees with the classical Gompertz model in the experiments by Sasaki et al. [36].

In the last set of simulations, we integrate the apoptosis signaling model into the working model of avascular tumor and analyze the growth under secretome treatment.  Figure 7 shows the correlation between secretome concentration and reduction in tumor volume as well as in the number of live cells. The results indicate the effectiveness of secretome in suppressing growth of avascular tumor.

With this result, our model provides an initial tool to predict the effect of MSC secretome in tumor growth both in cell culture and also in tumor spheroid experiment. Even though the model is comprehensive and encompassing, it still has several limitations. The first limitation is due to data unavailability of some parameter values. Hence, our simulations use estimated values that are not yet experimentally tested. Although this may not change the result that much, as verified by the sensitivity analysis, future studies can be done to obtain these parameter values by fitting the model to experimental data. The other limitation comes from the fact that it is a two-dimensional model and only captures tumor growth during avascular stage. For future work, we will extend the model to three dimensions for better accuracy and also simulate angiogenesis to study the effect of secretome during vascular growth.

## Figures and Tables

**Table 1:** Biochemical kinetics involved in apoptosis signaling pathways.

Extrinsic pathway	$Secretome+FasL\overset{k_{11}}{⟶}{FasL}^{*}$
	${FasL}^{*}+Casp8\overset{k_{12}^{+}}{\underset{k_{12}^{-}}{⇌}}{FasL}^{*}.Casp8\overset{k_{13}}{\to }{Casp8}^{*}$
	${Casp8}^{*}+Bid\overset{k_{51}^{+}}{\underset{k_{51}^{-}}{⇌}}{Casp8}^{*}.Bid\overset{k_{52}}{⟶}tBid$
	${Casp8}^{*}+Casp3\overset{k_{14}^{+}}{\underset{k_{14}^{-}}{⇌}}{Casp8}^{*}.Casp3\overset{k_{15}}{\to }{Casp8}^{*}+{Casp3}^{*}\overset{k_{16}}{⟶}Apoptosis$

Intrinsic pathway	$Secretome+DNA\overset{k_{21}}{⟶}DNAdamage$
	$DNAdamage+ATR\overset{k_{22}}{⟶}{ATR}^{*}$
	${ATR}^{*}+p53\overset{k_{23}^{+}}{\underset{k_{23}^{-}}{⇌}}{ATR}^{*}.p53\overset{k_{24}}{⟶}{p53}^{*}$
	${p53}^{*}+Bax\overset{k_{25}^{+}}{\underset{k_{25}^{-}}{⇌}}{p53}^{*}.Bax\overset{k_{26}}{⟶}Bax.Bak$
	$tBid+Bax\overset{k_{55}^{+}}{\underset{k_{55}^{-}}{⇌}}tBid.Bax\overset{k_{56}}{⟶}Bax.Bak$
	$Bcl2+{p53}^{*}\overset{k_{27}^{+}}{\underset{k_{27}^{-}}{⇌}}{p53}^{*}.Bcl2$
	$Bcl2+Bax\overset{k_{28}^{+}}{\underset{k_{28}^{-}}{⇌}}Bcl2.Bax$
	$Bax.Bak+Cytc_{mit}\overset{k_{29}}{⟶}Bax.Bak+Cytc$
	$Cytc+Casp9\overset{k_{30}^{+}}{\underset{k_{30}^{-}}{⇌}}Cytc.Casp9\overset{k_{31}}{⟶}{Casp9}^{*}$
	${Casp9}^{*}+Casp3\overset{k_{32}^{+}}{\underset{k_{32}^{-}}{⇌}}{Casp9}^{*}.Casp3\overset{k_{33}}{⟶}{Casp9}^{*}+{Casp3}^{*}\overset{k_{34}}{⟶}Apoptosis$

Perforin pathway	$Secretome+granB\overset{k_{41}}{⟶}{granB}^{*}$
	${granB}^{*}+Casp10\overset{k_{42}^{+}}{\underset{k_{42}^{-}}{⇌}}{granB}^{*}.Casp10\overset{k_{43}}{⟶}{Casp10}^{*}$
	${granB}^{*}+Bid\overset{k_{53}^{+}}{\underset{k_{53}^{-}}{⇌}}{granB}^{*}.Bid\overset{k_{54}}{⟶}tBid$
	${Casp10}^{*}+Casp3\overset{k_{44}^{+}}{\underset{k_{44}^{-}}{⇌}}{Casp10}^{*}.Casp3\overset{k_{45}}{⟶}{Casp10}^{*}+{Casp3}^{*}\overset{k_{46}}{⟶}Apoptosis$

A^*∗*^: the activated state of protein A; A · B: the compound of proteins A and B; *k* ^+^: forward rate constant of reaction; *k* ^−^: reverse rate constant of reaction; Cytc_mit  _: cytochrome c in mitochondria; Cytc: the released cytochrome c.

**Table 2:** The system of ordinary differential equations for the biochemical kinetics of the apoptosis signaling pathways. Blocks A, B, and C list the equations involved in extrinsic, intrinsic, and perforin pathways, respectively, and block D contains the equations used in all three pathways.

A	*d*[FasL^*∗*^]/*dt* = *k*_11_[Secretome][FasL] − *k*_12_^+^[FasL^*∗*^][Casp8] + *k*_12_^−^[FasL^*∗*^.Casp8]
	*d*[Casp8]/*dt* = −*k*_12_^+^[FasL^*∗*^][Casp8] + *k*_12_^−^[FasL^*∗*^.Casp8]
	*d*[FasL^*∗*^.Casp8]/*dt* = *k*_12_^+^[FasL^*∗*^][Casp8] − *k*_12_^−^[FasL^*∗*^.Casp8] − *k*_13_[FasL^*∗*^.Casp8]
	*d*[Casp8^*∗*^]/*dt* = *k*_13_[FasL^*∗*^.Casp8] − *k*_14_^+^[Casp8^*∗*^][Casp3] + *k*_14_^−^[Casp8^*∗*^.Casp3] + *k*_15_[Casp8^*∗*^.Casp3] − *k*_16_[Casp8^*∗*^][Casp3^*∗*^]
	−*k*_51_^+^[Casp8^*∗*^][Bid] + *k*_51_^−^[Casp8^*∗*^.Bid]
	*d*[Casp8^*∗*^.Bid]/*dt* = *k*_51_^+^[Casp8^*∗*^][Bid] − *k*_51_^−^[Casp8^*∗*^.Bid] − *k*_52_[Casp8^*∗*^.Bid]
	*d*[Casp8^*∗*^.Casp3]/*dt* = *k*_14_^+^[Casp8^*∗*^][Casp3] − *k*_14_^−^[Casp8^*∗*^.Casp3] − *k*_15_[Casp8^*∗*^.Casp3]

B	*d*[DNAdamage]/*dt* = *k*_21_[Secretome][DNA] − *k*_22_[DNAdamage][ATR]
	*d*[ATR^*∗*^]/*dt* = *k*_22_[DNAdamage][ATR] − *k*_23_^+^[ATR^*∗*^][p53] + *k*_23_^−^[ATR^*∗*^.p53]
	*d*[p53]/*dt* = −*k*_23_^+^[ATR^*∗*^][p53] + *k*_23_^−^[ATR^*∗*^.p53]
	*d*[ATR^*∗*^.p53]/*dt* = *k*_23_^+^[ATR^*∗*^][p53] − *k*_23_^−^[ATR^*∗*^.p53] − *k*_24_[ATR^*∗*^.p53]
	*d*[p53^*∗*^]/*dt* = *k*_24_[ATR^*∗*^.p53] − *k*_25_^+^[p53^*∗*^][Bax] + *k*_25_^−^[p53^*∗*^.Bax] + *k*_27_^−^[p53^*∗*^.BCl2] − *k*_27_^+^[BCl2][p53^*∗*^]
	*d*[Bax]/*dt* = −*k*_25_^+^[p53^*∗*^][Bax] + *k*_25_^−^[p53^*∗*^.Bax] − *k*_28_^+^[BCl2][Bax] + *k*_28_^−^[BCl2.Bax]
	−*k*_55_^+^[tBid][Bax] + *k*_55_^−^[tBid.Bax]
	*d*[p53^*∗*^.Bax]/*dt* = *k*_25_^+^[p53^*∗*^][Bax] − *k*_25_^−^[p53^*∗*^.Bax] − *k*_26_[p53^*∗*^.Bax]
	*d*[Bax.Bak]/*dt* = *k*_26_[p53^*∗*^.Bax] + *k*_56_[tBid.Bax]
	*d*[tBid.Bax]/*dt* = *k*_55_^+^[tBid][Bax] − *k*_55_^−^[tBid.Bax] − *k*_56_[tBid.Bax]
	*d*[BCl2]/*dt* = −*k*_27_^+^[BCl2][p53^*∗*^] + *k*_27_^−^[p53^*∗*^.BCl2] − *k*_28_^+^[BCl2][Bax] + *k*_28_^−^[BCl2.Bax]
	*d*[p53^*∗*^.BCl2]/*dt* = *k*_27_^+^[BCl2][p53^*∗*^] − *k*_27_^−^[p53^*∗*^.BCl2]
	*d*[BCl2.Bax]/*dt* = *k*_28_^+^[BCl2][Bax] − *k*_28_^−^[BCl2.Bax]
	*d*[Cytc_mit_]/*dt* = −*k*_29_[Bax.Bak][Cytc_mit_]
	*d*[Cytc]/*dt* = *k*_29_[Bax.Bak][Cytc_mit_] − *k*_30_^+^[Cytc][Casp9] + *k*_30_^−^[Cytc.Casp9]
	*d*[Casp9]/*dt* = −*k*_30_^+^[Cytc][Casp9] + *k*_30_^−^[Cytc.Casp9]
	*d*[Cytc.Casp9]/*dt* = *k*_30_^+^[Cytc][Casp9] − *k*_30_^−^[Cytc.Casp9] − *k*_31_[Cytc.Casp9]
	*d*[Casp9^*∗*^]/*dt* = *k*_31_[Cytc.Casp9] − *k*_32_^+^[Casp9^*∗*^][Casp3] + *k*_32_^−^[Casp9^*∗*^.Casp3] + *k*_33_[Casp9^*∗*^.Casp3] − *k*_34_[Casp9^*∗*^][Casp3^*∗*^]
	*d*[Casp9^*∗*^.Casp3]/*dt* = *k*_32_^+^[Casp9^*∗*^][Casp3] − *k*_32_^−^[Casp9^*∗*^.Casp3] − *k*_33_[Casp9^*∗*^.Casp3]

C	*d*[granB^*∗*^]/*dt* = *k*_41_[Secretome][granB] − *k*_42_^+^[granB^*∗*^][Casp10] + *k*_42_^−^[granB^*∗*^.Casp10] − *k*_53_^+^[granB^*∗*^][Bid] + *k*_53_^−^[granB^*∗*^.Bid]
	*d*[granB^*∗*^.Bid]/*dt* = *k*_53_^+^[granB^*∗*^][Bid] − *k*_53_^−^[granB^*∗*^.Bid] − *k*_54_[granB^*∗*^.Bid]
	*d*[Casp10]/*dt* = −*k*_42_^+^[granB^*∗*^][Casp10] + *k*_42_^−^[granB^*∗*^.Casp10]
	*d*[granB^*∗*^.Casp10]/*dt* = *k*_42_^+^[granB^*∗*^][Casp10] − *k*_42_^−^[granB^*∗*^.Casp10] − *k*_43_[granB^*∗*^.Casp10]
	*d*[Casp10^*∗*^]/*dt* = *k*_43_[granB^*∗*^.Casp10] − *k*_44_^+^[Casp10^*∗*^][Casp3] + *k*_44_^−^[Casp10^*∗*^.Casp3] + *k*_45_[Casp10^*∗*^.Casp3] − *k*_46_[Casp10^*∗*^][Casp3^*∗*^]
	*d*[Casp10^*∗*^.Casp3]/*dt* = *k*_44_^+^[Casp10^*∗*^][Casp3] − *k*_44_^−^[Casp10^*∗*^.Casp3] − *k*_45_[Casp10^*∗*^.Casp3]

D	*d*[Bid]/*dt* = −*k*_51_^+^[Casp8^*∗*^][Bid] + *k*_51_^−^[Casp8^*∗*^.Bid] − *k*_53_^+^[granB^*∗*^][Bid] + *k*_53_^−^[granB^*∗*^.Bid]
	*d*[tBid]/*dt* = *k*_52_[Casp8^*∗*^.Bid] + *k*_54_[granB^*∗*^.Bid] − *k*_55_^+^[tBid][Bax] + *k*_55_^−^[tBid.Bax]
	*d*[Casp3]/*dt* = −*k*_14_^+^[Casp8^*∗*^][Casp3] + *k*_14_^−^[Casp8^*∗*^.Casp3] − *k*_32_^+^[Casp9^*∗*^][Casp3] + *k*_32_^−^[Casp9^*∗*^.Casp3]
	−*k*_44_^+^[Casp10^*∗*^][Casp3] + *k*_44_^−^[Casp10^*∗*^.Casp3]
	*d*[Casp3^*∗*^]/*dt* = *k*_15_[Casp8^*∗*^.Casp3] − *k*_16_[Casp8^*∗*^][Casp3^*∗*^] + *k*_33_[Casp9^*∗*^.Casp3] − *k*_34_[Casp9^*∗*^][Casp3^*∗*^]
	+*k*_45_[Casp10^*∗*^.Casp3] − *k*_46_[Casp10^*∗*^][Casp3^*∗*^]
	*d*[Apop]/*dt* = *k*_16_[Casp8^*∗*^][Casp3^*∗*^] + *k*_34_[Casp9^*∗*^][Casp3^*∗*^] + *k*_46_[Casp10^*∗*^][Casp3^*∗*^]

**Table 3:** Reaction rate constants for biochemical kinetics used in the simulation.

Extrinsic pathway		Intrinsic pathway				Perforin pathway	
*k*_11_	0.5 *μ*M^−1^s^−1^	*k*_21_	0.5 *μ*M^−1^s^−1^	*k*_28_^+^	1 *μ*M^−1^s^−1^	*k*_41_^†^	0.5 *μ*M^−1^s^−1^
*k*_12_^+^	1 *μ*M^−1^s^−1^	*k*_22_	0.5 *μ*M^−1^s^−1^	*k*_28_^−^	1 s^−1^	*k*_42_^+^^†^	1 *μ*M^−1^s^−1^
*k*_12_^−^	1 s^−1^	*k*_23_^+^	1 *μ*M^−1^s^−1^	*k*_29_	10 *μ*M^−1^s^−1^	*k*_42_^−^^†^	1 s^−1^
*k*_13_	1 s^−1^	*k*_23_^−^	1 s^−1^	*k*_30_^+^	1 *μ*M^−1^s^−1^	*k*_43_^†^	1 s^−1^
*k*_14_^+^	1 *μ*M^−1^s^−1^	*k*_24_	1 s^−1^	*k*_30_^−^	1 s^−1^	*k*_44_^+^^†^	1 *μ*M^−1^s^−1^
*k*_14_^−^	1 s^−1^	*k*_25_^+^	1 *μ*M^−1^s^−1^	*k*_31_	1 s^−1^	*k*_44_^−^^†^	1 s^−1^
*k*_15_	1 s^−1^	*k*_25_^−^	1 s^−1^	*k*_32_^+^	10 *μ*M^−1^s^−1^	*k*_45_^†^	1 s^−1^
*k*_16_	1 *μ*M^−1^s^−1^	*k*_26_	1 s^−1^	*k*_32_^−^	0.5 s^−1^	*k*_46_^†^	1 *μ*M^−1^s^−1^
*k*_51_^+^^†^	1 *μ*M^−1^s^−1^	*k*_27_^+^	1 *μ*M^−1^s^−1^	*k*_33_	0.1 s^−1^	*k*_53_^+^^†^	1 *μ*M^−1^s^−1^
*k*_51_^−^^†^	1 s^−1^	*k*_27_^−^	1 s^−1^	*k*_34_	1 *μ*M^−1^s^−1^	*k*_53_^−^^†^	1 s^−1^
*k*_52_^†^	1 s^−1^	*k*_55_^+^^†^	1 *μ*M^−1^s^−1^	*k*_56_^†^	1 s^−1^	*k*_54_^†^	1 s^−1^
		*k*_55_^−^^†^	1 s^−1^				

†, estimated parameters. All other values are taken from [24]. The superscript “+” indicates forward rate constant and “−” reverse rate constant. The units for reaction rate constants are *μ*M^−1^s^−1^ for bimolecular reactions and s^−1^ for monomolecular reactions.

**Table 4:** Initial values of apoptosis proteins used in the simulation.

FasL	[0,1]	FasL^*∗*^	0	Casp9	[0,1]	Casp9^*∗*^	0
Casp8	[0,1]	Casp8^*∗*^	0	granB	[0,1]	granB^*∗*^	0
Casp3	[0,1]	Casp3^*∗*^	0	Casp10	[0,1]	Casp10^*∗*^	0
Apop	0			FasL^*∗*^.Casp8	0	Casp8^*∗*^.Casp3	0
Bid	[0,1]	tBid	0	ATR^*∗*^.p53	0	p53^*∗*^.Bax	0
DNA	[0,1]	DNAdamage	0	Bax.Bak	0	p53^*∗*^.BCl2	0
ATR	[0,1]	ATR^*∗*^	0	BCl2.Bax	0	Cytc.Casp9	0
p53	[0,1]	p53^*∗*^	0	Casp9^*∗*^.Casp3	0	granB^*∗*^.Casp10	0
Bax	[0,1]	BCl2	[0,1]	Casp10^*∗*^.Casp3	0	Casp8^*∗*^.Bid	0
Cytc_mit_	[0,1]	Cytc	0	tBid.Bax	0	granB^*∗*^.Bid	0

All values are in nondimensional form. The value [0,1] means a uniformly random number between 0 and 1. A^*∗*^: the activated state of protein A; A · B: the compound of proteins A and B; Cytc_mit_: cytochrome c in mitochondria; Cytc: the released cytochrome c.

**Table 5:** Parameter values used in the extracellular components of the model.

Symbol	Parameter	Value	Ref.
*D*_*u*_	Nutrient (oxygen) diffusion coefficient	0.00197 mm^2^/s	[32]
*γ*_*u*_	Single cell oxygen consumption rate	2.69 × 10^−17^ M/cells/s	[33]
*δ*_*u*_	Nutrient (oxygen) decay rate	0^*∗*^	Est.
*ϵ*	Degree of localized nutrient consumption	0.1	Est.
*D*_*m*_	MDE diffusion coefficient	10^−7^ mm^2^/s	[34]
*μ*	Single cell MDE production rate	1^*∗*^	[19]
*δ*_*m*_	MDE decay rate	0^*∗*^	[19]
*δ*_*f*_	ECM degradation rate	50^*∗*^	[19]
*D*_*w*_	Inhibitor (lactate) diffusion coefficient	1.67 × 10^−6^ mm^2^/s	[16]
*α*	Inhibitory factor production rate	2%/h/cm^3^	[16]
*T*_1_, *T*_2_	Nutrient threshold for necrosis and quiescent states	0.3, 0.4^*∗*^	Est.
*T*_3_	Growth inhibitor threshold	20^*∗*^	Est.

*∗*: nondimensionalized value.

**Table 6:** Parameter values used in the discrete component of the simulation.

Symbol	Parameter	Value	Ref.
*r*	Tumor cell radius	5–50 *μ*m	[35]
*C*_*A*_/*C*_*R*_	Attraction to repulsion coefficient ratio	0.3^*∗*^	[17]
*L*_*A*_, *L*_*R*_	Attraction and repulsion length scales	0.5, 0.1^*∗*^	[17]
*ξ*	Haptotaxis coefficient	2600 cm^2^/s/M	[19]
*T*	Duration of cell cycle	0.8–1.0 days (HeLa cell)	[36]

*∗*: nondimensionalized value.

**Table 7:** Percentage of apoptosis level contributed by each pathway for *t* = 48 hours.

Concentration	Total	Extrinsic	Intrinsic	Perforin	Cross-talk effect
0.2%	100%	0.64%	8.94%	2.08%	88.34%
2%	100%	3.55%	21.78%	9.04%	65.63%
20%	100%	35.01%	27.89%	59.00%	<0

All of these values come from Figures 4(a), 4(b), and 4(c).
